# A targeted illumination optical fiber probe for high resolution fluorescence imaging and optical switching

**DOI:** 10.1038/srep45654

**Published:** 2017-04-03

**Authors:** Anant Shinde, Sandeep Menon Perinchery, Vadakke Matham Murukeshan

**Affiliations:** 1 School of Mechanical and Aerospace Engineering, Nanyang Technological University, 639798 Singapore; 2 Centre for Optical and Laser Engineering, 50 Nanyang Avenue, 639798 Singapore

## Abstract

An optical imaging probe with targeted multispectral and spatiotemporal illumination features has applications in many diagnostic biomedical studies. However, these systems are mostly adapted in conventional microscopes, limiting their use for *in vitro* applications. We present a variable resolution imaging probe using a digital micromirror device (DMD) with an achievable maximum lateral resolution of 2.7 μm and an axial resolution of 5.5 μm, along with precise shape selective targeted illumination ability. We have demonstrated switching of different wavelengths to image multiple regions in the field of view. Moreover, the targeted illumination feature allows enhanced image contrast by time averaged imaging of selected regions with different optical exposure. The region specific multidirectional scanning feature of this probe has facilitated high speed targeted confocal imaging.

The optical systems with a targeted wavelength selective illumination and the ability to change the spatial pattern at high speeds have applications in different frontiers of science and technology such as biomedical imaging, optogenetics, selective filtering, microscopy and photodynamic therapy (PDT)^1^,^2^,^3^,^4^,^5^,^6^,^7^,^8^,^9^. Targeted illumination with a temporal precision of the order of milliseconds and the spatial precision of few tens of micrometers is a necessity for certain studies such as neuronal signalling^1^,^10^,^11^. Non-targeted illumination is also known to generate abrupt heat effects in regions of non-interests in many studies, hindering their research studies^6^,^12^,^13^. This elucidates the importance of targeted illumination system with an improved spatial and temporal resolution.

However, current targeted illumination systems are the adaptations of the conventional table top light microscopes^4^,^10^,^14^. These targeted illumination based microscopes are either limited by spatial resolution, temporal resolution or both^4^. Though there are multi-photon based imaging systems available with high spatial and temporal resolution, they are of a conventional desktop type and not clinician-friendly probe imaging systems^15^,^16^. Therefore designing a probe imaging system has incomparable advantage over other conventional microscopic systems.

In recent years, in addition to the conventional microscopic system, there are few fiber probe studies which have utilized standard structured illumination (grating patterns)^17^,^18^,^19^,^20^,^21^,^22^. However, high resolution fiber probes with targeted shape selective optical switching are not explored to their potential^19^,^23^,^24^,^25^,^26^,^27^. In this article, we have developed a novel, high-speed probe that can selectively (targeted) illuminate and manipulate any region in the sample space with microscale resolution. High resolution, targeted illumination, and optical switching features of the developed probe eliminate unwanted illumination of the sample regions in FOV. This probe enables to perform multi-wavelength illumination with different spatial patterns at temporal resolutions better than that of state of the art fiber probe systems. We have demonstrated the feasibility of the probe for the multidirectional subcellular targeted scanning and imaging by using mouse kidney sections as test samples. Moreover, we have also presented the enhancement in image contrast, by time averaged imaging of selected regions with different optical exposure and having different scanning patterns.

## Results

### Spatiotemporal illumination optical fiber probe

**Figure 1:**
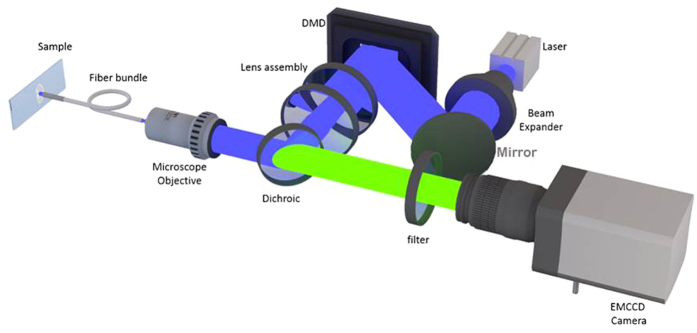
Targeted multispectral imaging fiber probe - Optical Configuration. Authors acknowledge Ruturaj Rajaram Patil for 3D rendering.

Figure 1 shows the schematic diagram representing the optical configuration of the developed fiber probe. A multiline laser combiner with the diode lasers of suitable wavelengths serves as the digitally controlled multispectral illumination source. The laser beam of 3 mm diameter is further expanded (5X magnification) with a beam expander [BE06R, Thorlabs] to cover the total area of the DMD mirror pane (14 mm × 10 mm). DMD [0.7 XGA DDR, Texas Instruments] is a reflective Spatial Light Modulator (SLM) with 1024 × 768 micromirrors; each micromirror (mirrorlets) is 13.7 μm × 13.7 μm in size and can be individually controlled to have the ‘ON’ or ‘OFF’ state of operation. An array of the DMD mirrorlets acts as a reflective grating creating the higher ordered diffraction beams. The zeroth order beam reflected from the DMD was allowed to pass through a condenser lens assembly (magnification 0.2X) of the plano convex lenses (focal length 35 mm and 75 mm) and a microscope objective lens (magnification 20X; NA 0.4 air objective) onto the proximal end of the fiber bundle. The laser beam is demagnified by a magnification factor 0.01X from DMD to fiber probe.

An imaging fiber bundle [Sumitomo Electric, IGN-11/50] of 1 mm diameter was used as the probe arm of the developed system. A custom gradient index (GRIN) lens [GT-IFRL-100-cus-50-NC, Grintech GmbH] having a focal length of 0.3 mm was attached to the distal end face of the fiber probe to enable non-contact imaging. The fiber bundle - GRIN lens assembly was secured in a steel sheath (1.1 mm inner diameter and 1.2 mm outer diameter). Microscope objective couple the light from the proximal end of the fiber bundle to Electron Magnified Charge Coupled Device (EMCCD) [iXon 887, Andor] camera. The bright field image collected through the fiber bundle is used as a reference input to select the regions in the sample to be illuminated. Using a commercial software [Pixel precision, 3DIcon], specific DMD mirrors were activated to perform targeted optical switching.

A multiline laser combiner with the laser sources of wavelengths 488 nm, 532 nm, and 561 nm is used as the excitation source which can illuminate simultaneously or sequentially the different fluorophores used in the study (Please see the sample preparation section).

### Multispectral spatiotemporal optical switching

**Figure 2:**
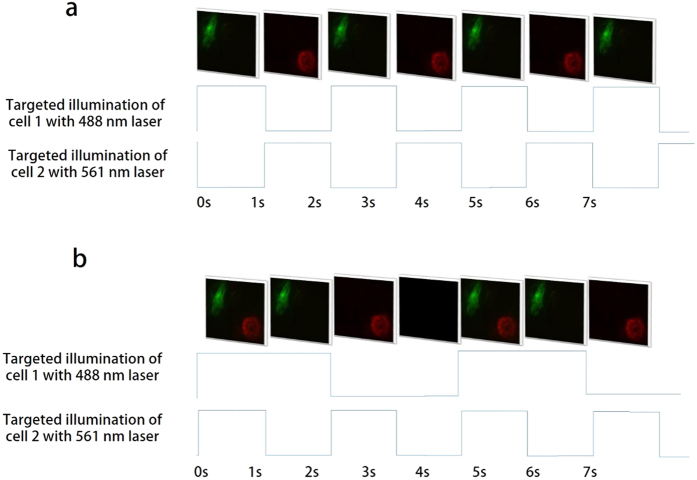
Multispectral targeted illumination switching of two cells from mouse kidney section. (**a**) Alternately target and illuminate cell 1 and cell 2 with an illumination wavelength of 488 nm and 561 nm respectively. (**b**) Illumination switching of 488 and 561 nm lasers to illuminate the targeted cells with different exposure times. The timing diagram showing laser ON and OFF time are provided for both (**a**) and (**b**).

The organelles in cells can respond differently to the individual wavelengths. It is reported that in certain scenarios, different organelles in the cells require light pulses of different wavelengths and exposures; therefore there is a need for systems with multispectral spatiotemporal optical switching capability^15^,^28^,^29^. Figure 2(a) shows the ability of the probe to selectively illuminate multiple cells or cell regions with different wavelengths (488 nm and 561 nm). The images of Fig. 2(a) are snapshots taken from Supplementary Video 1. In the Fig. 2, we used two laser beams which are timely separated in short time (0.001 s).

Additionally, Fig. 2(b) shows the selective multispectral optical switching at different locations in the same FOV using the probe. The optical switching speed of this probe is 10000 Hz which is limited by the DMD. Imaging speed of the Andor iXON EMCCD camera we used in our configuration is 25 fps. However for fluorescence imaging with low fluorescent concentration in the sample, a longer exposure time was needed to acquire the sufficient signal. Hence the optical switching is recorded at the low switching rate of 1 fps. The targeted optical switching feature using the probe is shown in Supplementary Video 2.

### Region specific scanning pattern

Scanning pattern is known to influence the image resolution of the system. Unlike the conventional microscopic or endoscopic imaging systems, which have a fixed scanning approach, the developed probe allows multiple regions to be scanned either simultaneously or at different timings with any pattern or in any direction with a spot size of 2.7 μm. As evident from Supplementary Video 3, the ROI can be scanned simultaneously using two fiberlets. Supplementary Video 4 demonstrate the probe’s ability to scan different regions with any type of illumination patterns.

### Confocal imaging with fiber probe

**Figure 3:**
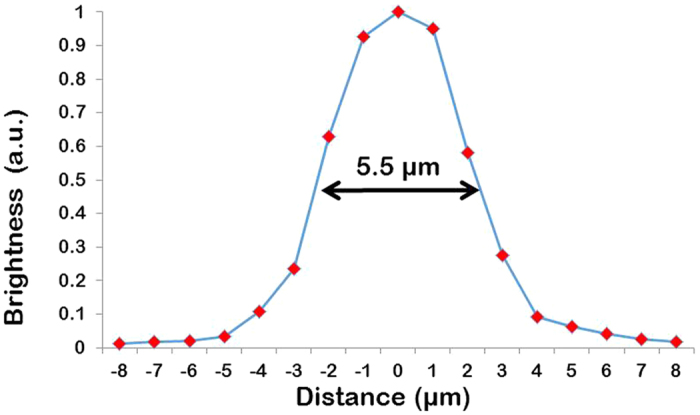
PSF of the developed fiber confocal imaging probe. (4 μm fluorescent beads, 561 nm excitation).

The pointwise scanning is an essential part of any confocal imaging system. In the developed fiber probe confocal imaging modality, scanning is achieved by illuminating one fiberlet at a time. Each fiberlet act as an individual pinhole and a scan point for fiber confocality. The information from each scan point is collected back through the same fiberlet and recorded as an image with the EMCCD camera. Locations of the fiberlets centers are used to reconstruct the confocal image^30^. FOV of the developed fiber probe is 140 μm × 100 μm. The size of the FOV can be varied based on the objective magnification and condenser lens assembly. The PSF of the system was measured with the fluorescent microspheres (4 μm) on a cover glass slide as the test target (Fig. 3). The system achieved axial resolution of 5.5 μm.

### Targeted imaging approach

**Figure 4:**
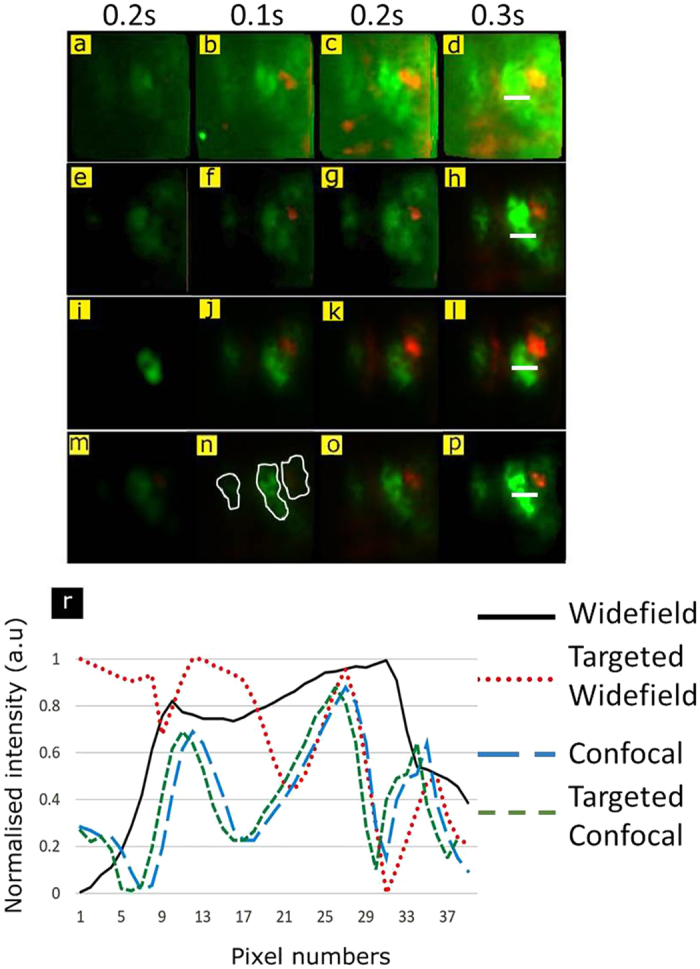
Different imaging schemes through the developed fiber probe. (**a,b,c**, and **d**) Widefield imaging **(e,f,g,** and **h**) confocal imaging (**i,j,k**, and **l**) Targeted widefield imaging and (**m,n,o,** and **p**)Targeted confocal imaging. Highlighted contours in (**n**) represent the three regions selected for targeted imaging (**r**) The intensity variation over the white line marked in images (**d**,**h**,**l**, and **p**).

Conventional fiber probe imaging methods use widefield and confocal approaches^24^,^31^. In this work, we have implemented targeted imaging approaches (In other words to image the regions of interest in FOV). To analyze effects of targeted imaging on image quality, we compared targeted imaging methods with the conventional fiber probe imaging methods (widefield and confocal). Figure 4 shows images acquired with the targeted and conventional fiber probe based methods with different exposure times. The regions selected for targeted imaging are marked in Fig. 4(n). Figure 4(r) depicts the intensity variation over a row of pixels as shown in images acquired with fiber probe based widefield, confocal, targeted widefield and targeted confocal methods. It is evident from the Fig. 4 that the targeted widefield imaging probe has better contrast (represented by intensity variation) compared to conventional fiber probe. In the case of confocal methods, the contrast values are comparable for conventional and targeted fiber probe.

The targeted confocal imaging is performed by simultaneously scanning multiple regions selected for targeted imaging. Figure 4(e–h) shows the conventional confocal images of the sample while Fig. 4(m–p) show the targeted confocal images of three selected regions. The time needed to acquire a targeted confocal image is controlled by the number of scan points in the largest of the three selected regions. Targeted confocal imaging demonstrated in Fig. 4 was 8 times faster than that of the conventional fiber confocal method. It is important to note that the imaging speed can vary based on the size of selected region and number of simultaneous scan points used.

### Targeted time averaged imaging

**Figure 5:**
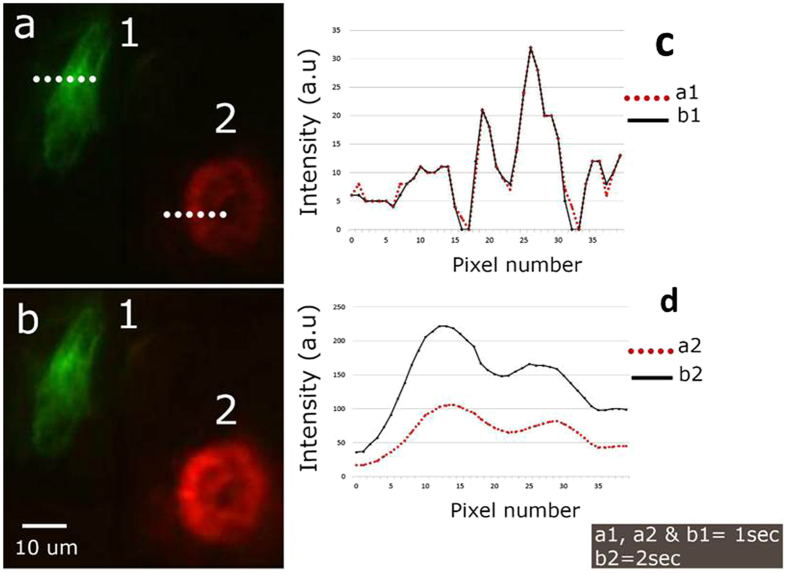
Targeted time averaged imaging of mouse kidney section. Cell 1 is illuminated with 488 nm, and cell 2 is illuminated with 561 nm. (**a**) Image acquired by illuminating cell 1 and cell 2 for 1 sec each. (**b**) Image acquired by illuminating cell 1 and cell 2 for 1 sec and 2 sec respectively. (**c** and **d**) Intensity variation across the line marked on cell 1 and cell 2 respectively.

The uneven concentration of fluorescence in sample always poses a hurdle in imaging with optimal quality. In conventional microscopy, time averaging (long exposure time) has to be used to capture a good quality image of the sample region with a low fluorescence concentration. On the contrary, for imaging the samples with high fluorescence concentration, the exposure time has to be reduced as well. These adjustments in the exposure times affect the image quality for rest of the image. In this context, we have developed a targeted time averaged imaging method. The region from the sample with low fluorescence concentration is selected and targeted time averaged imaging is performed. In this method other parts of the sample are imaged with smaller exposure time, providing an optimal image of the sample having uneven fluorescence concentration. Figure 5 shows images of cells (1 and 2) captured with different optical exposures. As evident from the Fig. 5, the targeted time averaged imaging improved the image quality without affecting rest of the image.

## Discussion

Table 1 shows relevant and reported fiber optic probes to perform high resolution imaging^18^,^20^,^32^,^33^,^34^. It is evident from the Table 1 that the probes based on specialty fiber optics used for targeted illumination, scanning, and optical switching is an underexplored area. The fiber probe presented in this manuscript has many potential advantages such as resolution improvement, wavelength switching option etc. as demonstrated. It has a lateral image resolution of 2.7 μm and an axial resolution of 5.5 μm, which is limited by the point spread function of the GRIN lens and diameter of the respective fiberlet. We envision that further improvements in the image resolution are possible with increasing the NA of GRIN lens and reduction in fiberlet diameter. Moreover, our system can perform targeted illumination of the subcellular structure with a resolution of 2.7 μm, which is better compared to the resolution achieved by earlier reported fiber probe systems (Table 1). The optical switching frequency of illumination pattern for our system is controlled by the DMD switching frequency. This can achieve a maximum switching frequency of 10000 Hz, which can find potential applications in neuronal signalling studies.

Experimental results have convincingly proven the viability of performing targeted illumination with the developed probe. This proposed concept and the developed probe have shown significant improvement in the signal contrast compared to conventional fiber probe based widefield imaging. Further, we have demonstrated the use of targeted confocal imaging approach which has shown substantial improvements in speed compared to conventional confocal imaging methods.

We have also presented the use of the probe for region specific scanning and it’s potential for optical switching. The multidirectional scanning in targeted regions is experimentally demonstrated by recording real time image sequences. It is believed that the region specific multidirectional scanning patterns and optical switching will open up new avenues of research and can find significant applications in optical microscopy and translational medicine.

Further, this novel targeted time averaged imaging concept can be used to obtain optimal images of samples having uneven fluorescence concentrations as well. It also helps in avoiding over exposure thereby reducing bleaching or damage to sample effectively.

In conclusion, a fiber probe with targeted illumination and optical switching control is designed, developed and demonstrated. The developed probe enables illumination of multiple regions of FOV for targeted time averaged imaging. Targeted time averaged imaging has shown to improve the image quality without affecting the rest of the image. Controlled switching with different time periods enables studies to address not only biological problems but also imaging problems from various engineering disciplines. We have shown a quantitative and qualitative improvement in the image quality and resolution for the images acquired with the developed fiber probe. It is envisaged that the invaluable advantages provided by this proposed concepts and demonstrated multispectral probe along with its specialty features can make a great impact in the related biomedical imaging and microscopy arena.

## Methods

### Fiberlet selection control and illumination

**Figure 6:**
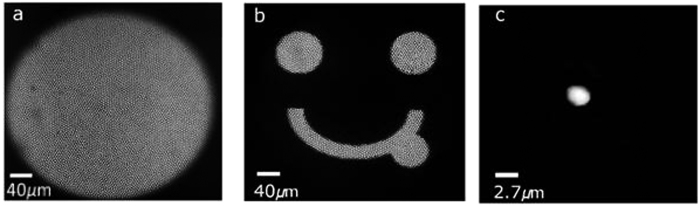
Selective illumination of fiber bundle end face. (**a**) End face of image fiber bundle with fiberlets clearly visible (with widefield illumination). (**b**) Representative spatial pattern illuminating the fiber bundle end face. (**c**) Selective illumination of one fiberlet of the fiber bundle (enlarged view).

For targeted illumination, it is important to couple the light through selected fiberlets so as to illuminate the target regions on the sample. Figure 6(a) shows the image of fiber bundle end face which clearly shows the fiberlets being illuminated. It is obtained with widefield illumination from the other end of the fiber bundle. Each fiberlet in the fiber bundle acts as a separate light conduit with negligible cross talks between the adjacent fibrelets. Moreover, the arrangement of fiberlets in the fiber bundle remains unchanged over its length, also known as coherent fiberlet arrangement. The fiberlet coherence and negligible cross talk preserve the selected spatial light pattern (illumination pattern) to propagate through the fiber bundle. Figure 6(b) shows the ability of probe to illuminate a sample with any predefined illumination pattern. Selective illumination through single fiberlet is illustrated in Fig. 6(c), it is the smallest possible illumination spot which can be illuminated with this probe having single fiberlet diameter of 2.7 μm.

**Figure 7:**
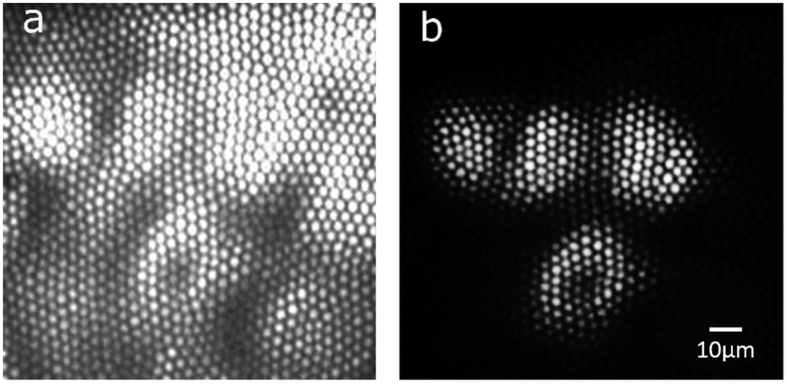
Comparison between fluorescence imaging with widefield and targeted illumination. (**a**) Image of mouse kidney section imaged with widefield illumination. (**b**) Targeted illumination and imaging cells from the same section.

Figure 6 has shown the ability of the probe to carry selective patterned illumination through the fiber bundle. This feature can be used to perform targeted illumination of the target regions of the sample. Initially, sample is imaged with widefield illumination. The illumination pattern is then modified to illuminate the target regions. Figure 7(a) shows the image of mouse kidney section imaged by illuminating the sample with widefield illumination. The illumination pattern is modified to illuminate the four target cells. The image of four cells from the FOV acquired with targeted illumination is shown in Fig. 7(b). Supplementary Video 5 demonstrates the selective illumination of a single cell, followed by targeted subcellular illumination. These results demonstrate the probe’s capability to perform a targeted illumination of the selected regions in the specimen.

### Image reconstruction

As the first step of image reconstruction, the mapping of DMD mirrorlets to the fiberlets has to be carried out. A bright field image of the white reflective surface was captured through the fiber bundle and used as a reference image. In the reference image, the center of each fiberlet was bright compared to the surrounding pixels. However, to find the exact positions of maxima, a Gaussian fitting was performed. Then 2D template and reference image was compared to identify the brightest locations for each fiberlet of the reference image. Further, the pixel locations of the fiberlet centers and the amplitude values were used to reconstruct the image devoid of pixelation noise by using interpolation method. The image reconstruction method used in this paper is further detailed in the ref. 30.

### Sample preparation

We used two test samples- first the fixed mouse kidney sections (Life Technologies, Inc.) and second the glass bubble sample.

Cells of the mouse kidney section were stained with fluorescent dyes Alexa Fluor 488 wheat germ agglutinin (ℷ_Ex Peak_ = 490 nm, ℷ_Em Peak_ = 525 nm) and Alexa Fluor 568 phalloidin (ℷ_Ex Peak_ = 578 nm, ℷ_Em Peak_ = 603 nm).

The Glass bubbles which were coated with Rhodamine 6G (**ℷ**_Ex Peak_ = 530 nm, **ℷ**_Em Peak_ = 566 nm) of known sizes were also used to study the sectioning ability of the fiber probe (Supplementary Video 3 and Supplementary Video 4). The sample was prepared using the glass bubble, the fluorescent dye and polyvinyl alcohol (PVA) in pure water. Initially, PVA was dissolved in warm water with the help of a magnetic stirrer. Following that glass bubbles (25 μm) and Rhodamine 6G were mixed in the solution, which was then transferred onto a glass slide and allowed to solidify.

## Additional Information

**How to cite this article**: Shinde, A. *et al*. A targeted illumination optical fiber probe for high resolution fluorescence imaging and optical switching. *Sci. Rep.* **7**, 45654; doi: 10.1038/srep45654 (2017).

**Publisher's note:** Springer Nature remains neutral with regard to jurisdictional claims in published maps and institutional affiliations.

## Supplementary Material

Supplementary Information

Supplementary Video 1

Supplementary Video 2

Supplementary Video 3

Supplementary Video 4

Supplementary Video 5

## Figures and Tables

**Table 1:** Comparison with state of the art fiber optic probes.

	Imaging Modalities			Targeted Illumination and imaging		Optical switching		Illumination scanning		Imaging Resolution (μm)	
Method	Widefield	SIM	Confocal	Spatial-TemporalMulti-target	Multispectral time averaged	Non-Targeted	Multi-targeted	Raster scanning	Region specific Scanning patterns	Spatial	Axial
Miniaturi zed fluorescence microscope^33^	✓									2.8	NA
SIM, HiLo Microendoscopy^18^,^32^	✓	✓								2.6	17
Pixelation-free and diffraction-limited imaging^34^,^35^	✓		✓					✓		1.09	8.99
Spatially Selective Holographic Photoactivation And Functional Fluorescence Imaging^20^	✓	✓	✓	✓		✓		✓		3.1	8.6
Probe method presented in this article	✓		✓	✓	✓	✓	✓	✓	✓	2.7	5.5
