# Supplementary material for: A targeted illumination optical fiber probe for high resolution fluorescence imaging and optical switching
Source: Sci Rep. 2017 Apr 3;7:45654. doi: 10.1038/srep45654 (PMC5377356; doi:10.1038/srep45654)
Supplement: Supplementary Information [file srep45654-s1.pdf]

A targeted illumination optical fiber probe for high resolution fluorescence imaging and optical switching

Anant Shinde[1,2], Sandeep Menon Perinchery[1,2] and Murukeshan Vadakke Matham[1,2]*

[1]School of Mechanical and Aerospace Engineering, Nanyang Technological University, Singapore 639798
[2]Centre for Optical and Laser Engineering, 50 Nanyang Avenue, Singapore 639798

*Address for Correspondence: mmurukeshan@ntu.edu.sg

Supplementary Video legends

Supplementary Video 1: Demonstration of multispectral spatiotemporal optical switching

Supplementary Video 2: Targeted optical switching using the developed fiber probe

Supplementary Video 3: Region specific scanning- example 1

Supplementary Video 4: Region specific scanning- example 2

Supplementary Video 5: Demonstration of fiberlet selection control and illumination with selective subcellular illumination.
